# Isolation of adult progenitor cells with neuronal potential from rabbit corneal epithelial cells in serum- and feeder layer-free culture conditions

**Published:** 2010-08-24

**Authors:** Tatsuya Mimura, Satoru Yamagami, Saiko Uchida, Seiichi Yokoo, Kyoko Ono, Tomohiko Usui, Shiro Amano

**Affiliations:** 1 Department of Ophthalmology, University of Tokyo Graduate School of Medicine, Tokyo, Japan; 2 Department of Ophthalmology, Toranomon Hospital, Tokyo, Japan; 3 Okinaka Memorial Institute for Medical Research, Tokyo, Japan; 4 Department of Ophthalmology, Tokyo Women's Medical University Medical Center East, Tokyo, Japan; 5 Corneal Regeneration Research Team Foundation for Biomedical research and Innovation, Kobe, Japan

## Abstract

**Purpose:**

To isolate progenitor cells from rabbit corneal epithelial cells (CEC) in serum- and feeder layer-free culture conditions and to compare the self-renewal capacity of corneal epithelial progenitor cells obtained from the central and limbal regions of the cornea.

**Methods:**

Tissue samples of New Zealand white rabbit corneas were dissected from the limbal and central regions to obtain CEC for sphere-forming culture, in which the cells formed spheres in serum-free medium containing growth factors. The number of primary and secondary sphere colonies and the size of the primary spheres were compared between the limbal and central regions. To promote differentiation, isolated sphere colonies were plated in dishes coated with poly-L-lysine (PLL)/laminin. The expression of epithelial, neural, and mesenchymal mRNAs was examined in the sphere colonies and their progeny by immunocytochemistry and/or the reverse transcription–polymerase chain reaction (RT–PCR). Adherent differentiated cells from the sphere colonies were also examined morphologically.

**Results:**

Primary spheres were isolated from both the limbal and central regions of the cornea. The rate of primary sphere formation by CEC from the limbal region (55.6±10.6/10,000 cells) was significantly higher than that by cells from the central cornea (43.1±7.2/10,000 cells, p=0.0028), but there was no significant difference in the size of primary spheres derived from both regions. The self-renewal capacity of cells from the limbal region was higher than that of cells from the central region, as evidenced by the significantly higher secondary sphere formation rate of limbal cells (38.7±8.5/10,000 cells) in comparison with that for central cells (31.3±5.7/10,000 cells, p=0.013). The primary sphere colonies expressed bromodeoxyuridine (BrdU), a 63-kDa protein (p63), p75 neurotrophin receptor (p75^NTR^), and nestin, whereas their progeny expressed cytokeratin 3, cytokeratin 12, vimentin, α*-*smooth muscle actin, microtubule-associated protein 2, and neuron-specific enolase on immunocytochemical analysis. These markers were confirmed by RT–PCR.

**Conclusions:**

Our findings indicate that limbal CEC contain more progenitor cells with a stronger self-renewal capacity than cells from the central region. These progenitor cells differentiate into the epithelial lineage, and can also produce neuronal protein.

## Introduction

The corneal epithelium (CE) is a nonkeratinized epithelium composed of multiple layers of cells with self-renewal capacity. Corneal epithelial stem cells are thought to be localized in the basal cell layer of the limbus and are believed to correspond to transient amplifying cells and terminally differentiated cells in the central CE [1-4]. Wound healing at the central CE occurs via centripetal migration and growth of stem cells from the periphery [5-9]. Over the past few years, a considerable number of workers have undertaken a molecular and histological analysis of corneal epithelial stem cells [10-16]. Kruse and Tseng [17] demonstrated that limbal stem cells could be differentiated into transient amplifying cells. Du et al. [18] reconstructed rabbit corneal surface using cultured human limbal cells on amniotic membrane. Yoshida et al. [19] demostrated neural crest-derived, multipotent stem cells exist in the adult cornea. However, little attention has been paid to attempting the selective isolation of stem cells or progenitor cells from the CE. Although corneal stem cells expressing neuronal markers were recently isolated from rodents [20], almost all of the differentiated cells derived from rodent corneal spheres show characteristics similar to those of fibroblast-like cells [21]. Thus, the separation of true epithelial spheres that retain the potential to differentiate into the corneal epithelial lineage has not been achieved yet because of the difficulty in isolating corneal epithelial cells (CEC) from mouse or rat stroma without contamination by fibroblasts. Because rabbit and human corneas are far larger than those of rats or mice, these may be more suitable for the isolation and characterization of corneal epithelial stem or progenitor cells.

In this study, we achieved the first isolation of adult progenitor cells from rabbit CE in serum- and feeder layer-free culture conditions. We also investigated the difference in self-renewal capacity between CEC from the central and limbal regions and whether the isolated cells could differentiate into multiple lineages.

## Methods

### Isolation of corneal epithelial cells

All animals were treated in accordance with the ARVO Statement on the Use of Animals in Ophthalmic and Vision Research and with the protocols approved by the Committee for Animal Research at the University of Tokyo Graduate School of Medicine. Rabbits were obtained from Saitama Experimental Animals Inc., Japan.

Primary cultures were established from 12-week-old male New Zealand white rabbits weighing an average of 2.4 kg. The basal medium for culture was DMEM/F12 (1:1; Sigma-Aldrich, St. Louis, MO) supplemented with 2% B27 (Invitrogen, San Diego, CA), 20 ng/ml of epidermal growth factor (EGF; Sigma-Aldrich), and 20 ng/ml of basic fibroblast growth factor (bFGF; Sigma-Aldrich), as described previously [22]. Anesthesia was induced by intramuscular injection of ketamine hydrochloride (60 mg/kg; Sankyo, Tokyo, Japan) and xylazine hydrochloride (10 mg/kg; Bayer, Leverkusen, Germany). After disinfection and sterile draping of the orbital region, a surgical blade was used to carefully dissect small tissue pieces from the limbal region. These tissue pieces measured approximately 1 mm×2 mm and were 100 μm thick with intact epithelium. To compare the sphere formation rate between the limbal cornea and central cornea, a sample of epithelium was excised from the central 6.0-mm region of the cornea. Each tissue sample was washed three times with sterile saline, and then immersed for 5 min in saline containing 10% povidone-iodine (Meiji, Tokyo, Japan) and 50 µg/ml gentamicin (Sigma-Aldrich). After further rinsing with saline, the limbal epithelial tissues were cut into small pieces and incubated in basal medium containing 0.02% collagenase (Sigma-Aldrich) overnight at 37 °C. After washing with Ca^2+^- Mg^2+^-free phosphate-buffered saline (PBS; Sigma-Aldrich), the tissue pieces were incubated in 0.2% EDTA at 37 °C for 5 min and were dissociated into single cells by trituration with a fire-polished Pasteur pipette. After centrifugation at 800× g for 5 min, the cells were resuspended in basal medium. Isolated limbal epithelial cells were counted with a Coulter counter and their viability was confirmed to be >90% by trypan blue staining (Wako Pure Chemical Industries, Osaka, Japan).

### Isolation of sphere colonies from corneal epithelial cells

Primary culture was done according to the neurosphere assay [23]. Basal medium containing methylcellulose gel matrix (1.5%; Wako Pure Chemical Industries) was employed to prevent reaggregation of the cells, as described previously [24]. Plating was done for floating culture at a density of 10.0 viable cells/μl (40,000 cells/well) in uncoated 60-mm culture dishes (BD Biosciences, San Jose, CA). Under these conditions, reaggregation did not occur and most (or all) of the sphere colonies were derived from single cells [24,25]. Culture was done in a humidified incubator with an atmosphere of 5% CO_2_.

After seven days, cell clusters (i.e., sphere colonies) were detected. The number of primary spheres was counted after 7 days of culture. To distinguish growing spheres from dying cell clusters, only clusters with a diameter >50 μm were counted. For passaging, primary spheres (day 7) were treated with 0.5% EDTA and dissociated into single cells, which were plated in 24-well culture plates at a density of 10.0 cells/μl. Then culture was done for a further 7 days in basal medium containing methylcellulose gel matrix. To measure the diameter of the sphere colonies, cultures were observed under an inverted phase-contrast microscope (Nikon ELWD 0.3, Tokyo, Japan) with a 10× objective lens, and the images were analyzed by employing the NIH image program developed at the USA National Institutes of Health and available on the Internet.

### Differentiation of sphere colonies

To assess the mulitipotentiality of isolated sphere colonies, individual primary spheres (day 7) were transferred to 13-mm glass coverslips coated with 50 μg/ml poly-L-lysine (PLL; Sigma-Aldrich) and 10 μg/ml fibronectin (BD Biosciences, Billerica, MA) in separate wells, as described previously [26]. To promote differentiation, 1% fetal bovine serum (FBS) was added to the basal medium to form differentiation medium and culture was continued for another 7 days.

### Immunohistochemistry of spheres for bromodeoxyuridine (BrdU)

Expression of BrdU in the sphere colonies was determined by immunocytochemistry. The 7-day primary spheres were incubated overnight with 10 μM/ml BrdU (Sigma-Aldrich). After fixing in methanol (Wako Pure Chemical Industries) at 4 °C for 5 min and treatment with 2 M HCl (Wako Pure Chemical Industries) in PBS at room temperature for 60 min, the cells from sphere colonies were stained with FITC-conjugated anti-BrdU antibody at room temperature for 60 min in the dark. After washing with PBS, fluorescence was observed under a fluorescence microscope (model BH2-RFL-T3 and BX50; Olympus, Tokyo, Japan).

### Immunocytochemistry of spheres and their progeny

Immunocytochemical analysis was performed on 7-day spheres and their progeny after 7 days of adherent culture on glass coverslips. Cells were fixed with 4% paraformaldehyde (Wako Pure Chemical Industries) in PBS for 10 min. After washing in PBS, the cells were incubated for 30 min with 4% BSA (BSA; Sigma-Aldrich) in PBS containing 0.3% Triton X-100 (BSA/PBST; Rohm & Haas, Philadelphia, PA) to block nonspecific binding. Then the cells were incubated for 2 h at room temperature with specific primary antibodies diluted in BSA/PBST. The following antibodies were used: mouse anti-cytokeratin 3 monoclonal antibody (1:2,000; AE-5; Progen Biotechnik GMBH, Heidelberg, Germany), goat anti-cytokeratin 12 polyclonal antibody (1:2,000; L-20; Santa Cruz Biotech, Santa Cruz, CA), mouse monoclonal anti-p63 antibody (1:400; Imgenex, San Diego, CA), mouse monoclonal anti-nestin antibody (1:400; BD Biosciences), mouse monoclonal anti-nerve growth factor (NGF) receptor p75^NTR^ antibody (1:400; DAKO, Kyoto, Japan), Cy3-conjugated mouse anti-α-smooth muscle actin (SMA) mAb (1:400; Sigma-Aldrich), mouse monoclonal anti-nestin antibody (1:400; BD Biosciences), mouse monoclonal anti- microtubule-associated protein 2 antibody (MAP2, 1:400; Chemicon, Temecula, CA), mouse monoclonal anti- neuron specific enolase antibody (NSE, 1:400; DAKO), and mouse monoclonal anti-BrdU/fluorescein antibody (1:100; Roche Diagnostics, Basel, Switzerland). As a negative control, mouse IgG (1:1,000, Sigma-Aldrich) was used instead of the primary antibody. After incubation with these primary antibodies, cells were incubated for 30 min with fluorescence-labeled anti-mouse IgG or anti-goat IgG (Alexa Fluor, 1:2,000; Molecular Probes, Eugene, OR) as the secondary antibody. After washing with PBS, fluorescence was observed under a fluorescence microscope.

### Extraction of total RNA and RT–PCR

Total RNA was isolated from primary sphere colonies, the adherent progeny of sphere colonies, and rabbit corneal epithelial cells before culture with a kit (Isogen; Nippon Gene, Tokyo, Japan) according to the manufacturer’s instructions, after which RT–PCR was done to investigate the expression of nestin, keratin-3, and glyceraldehyde-3-phosphate dehydrogenase (*G3PDH*) as a housekeeping gene. Then the isolated total RNA was treated with RNase-free DNase I (Stratagene, La Jolla, CA) for 30 min, and cDNA was formed by using Super Script II (Invitrogen) as the reverse transcriptase. T12VN primer (at a concentration of 25 ng/µl) was used to make the 1st strand. As the negative control, RT–PCR was performed in the absence of reverse transcriptase. The PCR buffer contained 1.5 mM MgCl_2_ with 0.2 mM of each dNTP (Applied Biosystems, Foster City, CA), 0.2 mM of each primer, and 25 units/l of Amp Taq Gold (Applied Biosystems). After an initial 9 min of denaturation at 95 °C, amplification was performed for 30 cycles (30 s at 94 °C, 30 s at 60 °C, and 45 s at 72 °C), followed by a final 7 min of elongation using a thermal cycler (I-Cycler; Bio Lad Laboratories, Hercules, CA). The oligonucleotide primers for RT–PCR were based on the sequences of *p63*, nestin, ketarin-3, keratin-12, and *G3PDH*. The primer pairs and produce size are shown in Table 1. Products were separated on 1% agarose gel and then visualized by staining with ethidium bromide.

**Table 1:** Primers used for polymerase chain reaction.

**Gene**	**Forward primer**	**Reverse primer**	**Size (bp)**
*p63*	5′-GCCACCTGGACGTATTCCAC-3′	5′-CATACTGGGCATGGCTGTTCC-3′	259
nestin	5′-TTGAGAC(A/T)CCTGTG(C/A)CAGCCT-3′	5′-CTCTAGAC(T/C)CAC(T/C)GGATTCT-3′	383–387
ketarin-3	5′-GCAGCAGCAGGATGAGCTG-3′	5′-GTTGAGGGTCTTGATCTG-3′	482
keratin-12	5′-GAGCTGGCCTACATGAAG-3′	5′-TTGCTGGACTGAAGCTGCTC-3′	250
*G3PDH*	5′-CATCACCATCTTCCAGGAGC-3′	5′-ACAATGCCGAAGTGGTCGTG-3′	294

### Statistics

Student’s unpaired *t*-test was used to compare mean values. All analyses were done with the Stat View statistical software package (Abacus Concepts, Berkeley, CA) and p<0.05 was considered to indicate significance.

## Results

### Isolation of sphere colonies from single cells

**Figure 1:**
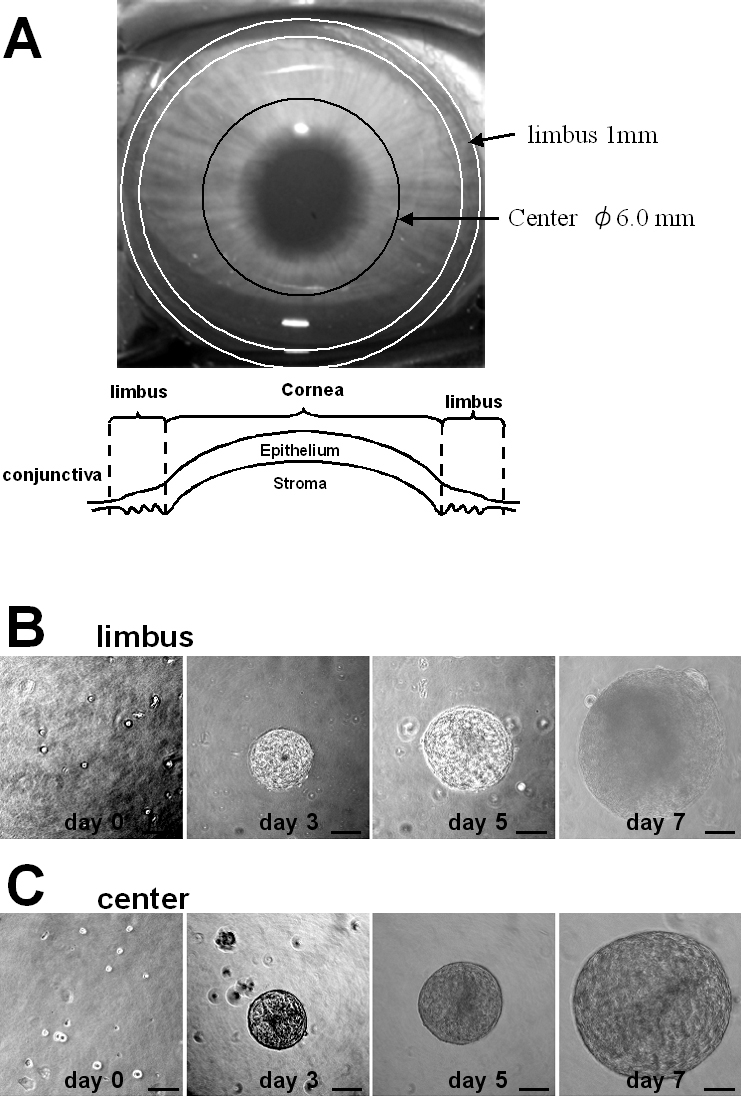
Sphere-forming culture of rabbit corneal epithelium. **A**: Anterior view of a rabbit cornea and a diagram of the corneal epithelium. **B**, **C**: Rabbit corneal epithelial cells from the limbus or central cornea formed spheres. Growth of a representative single sphere is shown until 7 day.

**Figure 2:**
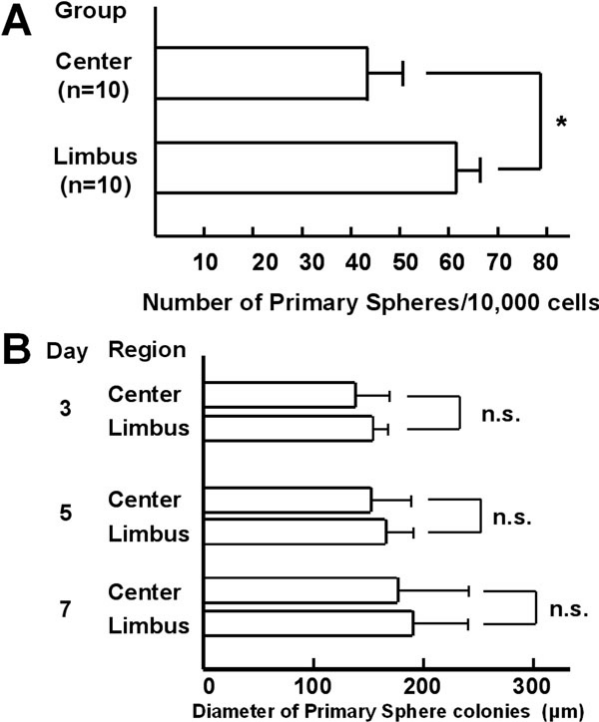
Comparison of primary sphere formation by rabbit CEC from the periphery and center of the cornea. **A**: The number of spheres obtained from limbal CEC (n=10) was significantly higher than that from central CEC (n=10) after 7 days of culture. The experiment was repeated twice and representative data are shown. The asterisk indicates a p=0.0028 (unpaired *t*-test). **B**: The mean sphere size exceeded 250 μm on day 7.

We adapted the neurosphere-forming assay that was originally devised to enrich neural stem cells and other progenitors [20,21,24,25,27-32] for isolation of adult stem cells from rabbit corneal limbal and central epithelium (Figure 1A). CEC were disaggregated into single cells and plated in uncoated wells with basal medium containing methylcellulose gel matrix to prevent reaggregation at a density of 10 viable cells/μl, as described elsewhere [24,25]. Under these conditions, sphere colonies are derived from proliferation and are not formed by reaggregation of dissociated cells [24,25]. Almost complete disaggregation into single cells was confirmed by counting the percentage of single cells, double cells, and triple cells, which demonstrated that more than 99% of the cells were single. Primary spheres were isolated from CEC derived from both the limbal and central regions. Photographs of representative spheres obtained from the limbal and central regions are shown in Figure 1B,C. When the number of sphere colonies obtained from the CEC was counted, there was a significantly greater number of spheres (55.6±10.6, mean±standard deviation) obtained from the limbal region than from the central region (43.1±7.2) per 10,000 plated cells (Figure 2A). There were no statistically significant differences in the size of primary spheres from the two regions after 3, 5, and 7 days (Figure 2B).

### Secondary sphere formation from primary spheres

**Figure 3:**
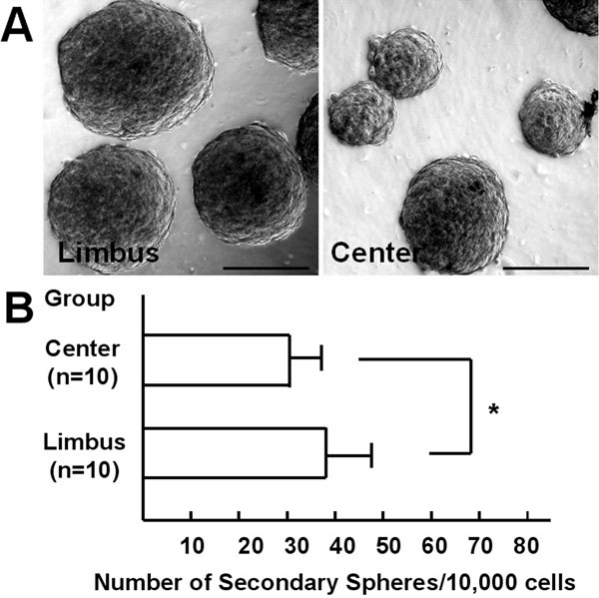
Formation of secondary spheres. **A**: Secondary spheres were generated from dissociated primary limbal or central spheres. **B**: The replating efficiency for formation of secondary spheres was higher when the cells were derived from the limbal cornea than from the central cornea (p=0.013, unpaired *t*-test).

To evaluate the self-renewal capacity of CEC, primary spheres were passaged under the same culture conditions as CEC. Secondary spheres were generated from dissociated primary spheres obtained from the limbal or central CEC. Photographs of representative secondary spheres are shown in Figure 3A. The number of secondary spheres per 10,000 cells was significantly higher for primary spheres from the limbal region than from the central region (38.7±8.5 versus 31.3±5.7, respectively, p=0.013, unpaired *t*-test; Figure 3B).

### Immunocytochemistry and RT–PCR of spheres

**Figure 4:**
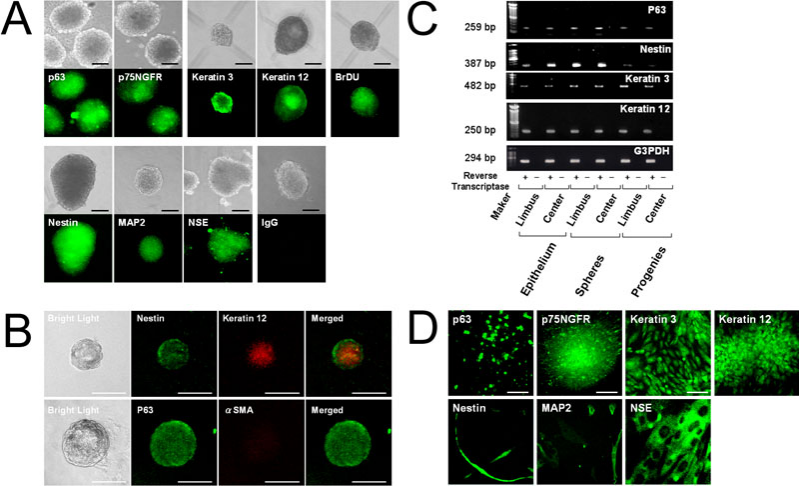
Analysis of spheres and their progeny. **A**: Immunocytochemical analysis of sphere colonies on day 7. Bright-field images and immunostaining of spheres for p63 (an epidermal stem/progenitor cell marker), p75^NTR^ (an epidermal basal progenitor cell marker), cytokeratins 3 and 12 (differentiated epithelial cell markers, nestin (a neural stem cell marker), microtubule-associated protein 2 (MAP2: a differentiated neuronal cell marker), and neuron-specific enolase (NSE: a differentiated neuronal cell marker). Each colony has been labeled by BrdU. As a control, IgG was used instead of the primary antibody. Scale bar=100 µm. **B**: Double immunocytochemical staining of a sphere colony. The spheres are double immunostained by nestin and cytokeratin 12 or by p63 and alpha smooth muscle actin (αSMA). Scale bar=100 µm. **C**: RT–PCR of corneal epithelial tissues, spheres, and their progeny. Genes for *P63*, keratin 3, keratin 12, and nestin are present in corneal epithelial tissues, spheres, and their progeny derived from the limbal or central regions, but not in total RNA processed without reverse-transcription. **D**: Immunocytochemical analysis of differentiated cells obtained from spheres. Cells migrating out of the spheres express both cytokeratin 3 and cytokeratin 12 (differentiated epithelial cell markers). These cells are also positive for MAP2, and NSE. Scale bar=100 µm.

Nuclear protein p63 was recently proposed as a progenitor cell marker that can be used to identify epidermal stem cells [33]. Nestin is expressed by immature neural progenitor cells in multipotential sphere colonies derived from the brain [34], skin [22], inner ear [35], retina [36], corneal stroma [27], and endothelium [28-32]. To examine the potential of sphere colonies, primary spheres were immunostained for p63 and nestin as stem/progenitor cell markers. Additionally, p75 neurotrophin receptor (p75NTR), the nerve growth factor receptor, was used as a marker of epidermal basal progenitor cells [11]. Most cells in the spheres were immunoreactive for p63, p75^NTR^, nestin, and BrDU (Figure 4A,B). The expression of p63 and nestin was also confirmed by the detection of mRNA (Figure 4C). Spheres derived from both the limbal and central regions showed the same patterns of immunostaining and mRNA expression.

### Immunocytochemistry of the progeny of individual spheres

To investigate whether the progeny of the spheres possessed the characteristics of mesenchymal or neural lineage cells, single spheres (day 10 of culture) were transferred onto poly L lysine/fibronectin-coated glass coverslips in 24-well plates, and then were cultured in differentiation medium containing 1% fetal bovine serum (FBS). After 7 days, many cells migrated out from the spheres. A small population of these migrating cells was p63- and nestin-positive, while most of the cells were positive for cytokeratin 3, a differentiated epithelial cell marker. The cells were also positive for MAP2 and NSE, indicating that the cells showed neuronal differentiation under these conditions (Figure 4D). Thus, culture of CEC generated p63- and nestin-positive progenitor cells that gave rise to epidermal and neuronal cells, suggesting the bipotency of these progenitor cells.

## Discussion

Considerable attention has been directed toward understanding the role of stem cells or progenitor cells in corneal development. Kruse and Tseng [17] demonstrated the differentiation of isolated corneal stem cells to transient amplifying cells. Yoshida et al. [19,21] isolated corneal precursors from the mouse cornea by sphere-forming assay. They found that the phenotype of mouse keratocytes can be maintained in vitro for more than 12 passages by the serum-free sphere culturing technique [21] and that neural crest-derived, multipotent stem cells exist in the adult cornea [19]. However, there have been few reports about the harvesting of stem cells or progenitor cells from the cornea by sphere-forming culture, and the only research concerning the isolation of tissue-specific stem cells from the corneal limbal epithelium by this method has been performed in rodents [20]. In our previous study, CEC isolated from the human corneal limbus did not form spheres in floating culture and became adherent to uncoated culture dishes [37]. This suggests that rabbit CEC may be fundamentally different from human cells. In addition, we have previously reported on the isolation of corneal progenitor cells from the corneal stroma [27] and endothelium by sphere-forming culture [28-32]. We found that the cells isolated from adult rabbit corneal epithelium were directed toward the ectodermal lineages and expressed markers for epidermal, and neural cells. To our knowledge, this is the first report about differentiation of progenitor cells isolated from the limbal region or the central cornea into epidermal and neural cells by floating culture.

In this study, we isolated progenitor cells from rabbit CEC by the sphere-forming culture method established by Toma et al. [22]. Spheres derived from rabbit CEC had a strong proliferative capacity as shown by BrdU staining and were positive for the epidermal progenitor cell marker p63, the epidermal basal progenitor cell marker p75^NTR^, and the neuron stem cell marker nestin. Self-renewing potential was indicated by the ability of the progeny of individual spheres to generate secondary spheres. Regarding the self-renewal capacity of these cells, we could not exclude the possibility that these progenitor cells did not have a uniform renewal capacity, with all of the growth coming from a rare subset of cells in the spheres. Additional studies will be required to determine whether or not these progenitor cells are adult stem cells.

It is also noteworthy that the spheres were positive for CEC markers (cytokeratins 3 and 12), and a neuronal marker (MAP2), indicating that epithelial and neuronal differentiation occurred in the spheres under serum-free culture conditions. Moreover, the progeny of the spheres also expressed epithelial and neuronal markers. The neurotrophin receptor p75^NTR^ is most highly expressed in the progenitor cells of the corneal limbal epithelium [38]. Grueterich et al. [11] reported that p75^NTR^ was localized in the suprabasal limbal epithelium and entire corneal epithelium, but was not detected in the limbal basal epithelium, suggesting that p75NTR can be considered a differentiated progenitor marker of corneal epithelium. These findings indicate that the spheres contained lineage-uncommitted bipotent progenitor cells.

Various studies have already been conducted on corneal epithelial stem cells by diverse methods [10-16,39]. A common limitation of studies on these cells has been the stem cell isolation procedure. Zhao et al. [20] offered a model for characterizing the neural potential of corneal stem cells by the sphere-forming assay. However, they failed to show that CEC stem cells can differentiate into the authentic epithelial lineage. In this study, we proposed the first successful isolation procedure for progenitor cells that mainly generated CECs positive for cytokeratins 3 and 12. Thus, precursors obtained from the corneal epithelium may be more appropriate than multipotential stem cells for tissue regeneration or cell transplantation, because such precursors should efficiently differentiate to produce their tissue of origin.

We demonstrated that the number of spheres derived from the limbal epithelium was significantly higher than that obtained from the central corneal epithelium, while there was no significant difference in the size of spheres from the limbal and central areas. The expression of differentiation markers such as a specific corneal marker (cytokeratin 3) was markedly higher in spheres derived from the central cornea compared with spheres from the limbal cornea (data not shown), indicating that cells composing spheres derived from the central corneal epithelium were more prone to undergo differentiation. These findings imply that limbal epithelium contains more stem or progenitor cells than the central epithelium and that spheres derived from limbal CEC proliferate while maintaining a more immature state in comparison with CEC from the central cornea. Conversely, even the transient amplifying cells or progenitor cells of the rabbit central cornea may have a finite proliferative potential and may differentiate into the epithelial and neuronal lineages. The present findings obtained by sphere-forming culture are consistent with the concept that the corneal limbus is rich in stem cells [7-9].

In summary, we isolated adult progenitor cells from rabbit corneal epithelium by sphere-forming culture and compared the relative abundance and self-renewal capacity of CEC progenitor cells obtained form the central and limbal regions of the cornea. Our findings demonstrated that both limbal and central CEC contain a significant number of progenitor cells, although the limbal region of the rabbit cornea has a higher density of progenitor cells with a stronger self-renewal capacity than the central region. Future our study will focus on the transplantation of autologous isolated sphere-forming progenitors. Proliferating sphere-forming cells derived from CEC may be useful for the treatment of corneal diseases associated with limbal stem cell deficiency.

## References

[1] Potten CS Schofield R Lajtha LG A comparison of cell replacement in bone marrow, testis and three regions of surface epithelium. Biochim Biophys Acta 1979 560 281 99 380653 10.1016/0304-419x(79)90022-2

[2] Lavker RM Sun TT Heterogeneity in epidermal basal keratinocytes: morphological and functional correlations. Science 1982 215 1239 41 7058342 10.1126/science.7058342

[3] Lavker RM Sun TT Epidermal stem cells. J Invest Dermatol 1983 81 121s 7s 6190957 10.1111/1523-1747.ep12540880

[4] Ferraris C Chevalier G Favier B Jahoda CA Dhouailly D Adult corneal epithelium basal cells possess the capacity to activate epidermal, pilosebaceous and sweat gland genetic programs in response to embryonic dermal stimuli. Development 2000 127 5487 95 11076768 10.1242/dev.127.24.5487

[5] Kinoshita S Friend J Thoft RA Sex chromatin of donor corneal epithelium in rabbits. Invest Ophthalmol Vis Sci 1981 21 434 41 7024181

[6] Kinoshita S Kiorpes TC Friend J Thoft RA Limbal epithelium in ocular surface wound healing. Invest Ophthalmol Vis Sci 1982 23 73 80 7085223

[7] Thoft RA Friend J The X, Y, Z hypothesis of corneal epithelial maintenance. Invest Ophthalmol Vis Sci 1983 24 1442 3 6618809

[8] Schermer A Galvin S Sun TT Differentiation-related expression of a major 64K corneal keratin in vivo and in culture suggests limbal location of corneal epithelial stem cells. J Cell Biol 1986 103 49 62 2424919 10.1083/jcb.103.1.49 PMC2113783

[9] Zieske JD Bukusoglu G Yankauckas MA Characterization of a potential marker of corneal epithelial stem cells. Invest Ophthalmol Vis Sci 1992 33 143 52 1730535

[10] Grueterich M Espana EM Tseng SC Modulation of keratin and connexin expression in limbal epithelium expanded on denuded amniotic membrane with and without a 3T3 fibroblast feeder layer. Invest Ophthalmol Vis Sci 2003 44 4230 6 14507866 10.1167/iovs.02-0943

[11] Grueterich M Espana EM Tseng SC Ex vivo expansion of limbal epithelial stem cells: amniotic membrane serving as a stem cell niche. Surv Ophthalmol 2003 48 631 46 14609709 10.1016/j.survophthal.2003.08.003

[12] Espana EM Kawakita T Romano A Di Pascuale M Smiddy R Liu CY Tseng SC Stromal niche controls the plasticity of limbal and corneal epithelial differentiation in a rabbit model of recombined tissue. Invest Ophthalmol Vis Sci 2003 44 5130 5 14638708 10.1167/iovs.03-0584

[13] Chen Z de Paiva CS Luo L Kretzer FL Pflugfelder SC Li DQ Characterization of putative stem cell phenotype in human limbal epithelia. Stem Cells 2004 22 355 66 15153612 10.1634/stemcells.22-3-355 PMC2906385

[14] de Paiva CS Chen Z Corrales RM Pflugfelder SC Li DQ ABCG2 transporter identifies a population of clonogenic human limbal epithelial cells. Stem Cells 2005 23 63 73 15625123 10.1634/stemcells.2004-0093 PMC2906389

[15] Zhang X Sun H Tang X Ji J Li X Sun J Ma Z Yuan J Han ZC Comparison of cell-suspension and explant culture of rabbit limbal epithelial cells. Exp Eye Res 2005 80 227 33 15670801 10.1016/j.exer.2004.09.005

[16] Shurman DL Glazewski L Gumpert A Zieske JD Richard G In vivo and in vitro expression of connexins in the human corneal epithelium. Invest Ophthalmol Vis Sci 2005 46 1957 65 15914609 10.1167/iovs.04-1364

[17] Kruse FE Tseng SC A serum-free clonal growth assay for limbal, peripheral, and central corneal epithelium. Invest Ophthalmol Vis Sci 1991 32 2086 95 1711516

[18] Du Y Chen J Funderburgh JL Zhu X Li L Functional reconstruction of rabbit corneal epithelium by human limbal cells cultured on amniotic membrane. Mol Vis 2003 9 635 43 14685149 PMC2877914

[19] Yoshida S Shimmura S Nagoshi N Fukuda K Matsuzaki Y Okano H Tsubota K Isolation of multipotent neural crest-derived stem cells from the adult mouse cornea. Stem Cells 2006 24 2714 22 16888282 10.1634/stemcells.2006-0156

[20] Zhao X Das AV Thoreson WB James J Wattnem TE Rodriguez-Sierra J Ahmad I Adult corneal limbal epithelium: a model for studying neural potential of non-neural stem cells/progenitors. Dev Biol 2002 250 317 31 12376106

[21] Yoshida S Shimmura S Shimazaki J Shinozaki N Tsubota K Serum-free spheroid culture of mouse corneal keratocytes. Invest Ophthalmol Vis Sci 2005 46 1653 8 15851565 10.1167/iovs.04-1405

[22] Toma JG Akhavan M Fernandes KJ Barnabe-Heider F Sadikot A Kaplan DR Miller FD Isolation of multipotent adult stem cells from the dermis of mammalian skin. Nat Cell Biol 2001 3 778 84 11533656 10.1038/ncb0901-778

[23] Irvine AD Corden LD Swensson O Swensson B Moore JE Frazer DG Smith FJ Knowlton RG Christophers E Rochels R Uitto J McLean WH Mutations in cornea-specific keratin K3 or K12 genes cause Meesmann's corneal dystrophy. Nat Genet 1997 16 184 7 9171831 10.1038/ng0697-184

[24] Gritti A Frolichsthal-Schoeller P Galli R Parati EA Cova L Pagano SF Bjornson CR Vescovi AL Epidermal and fibroblast growth factors behave as mitogenic regulators for a single multipotent stem cell-like population from the subventricular region of the adult mouse forebrain. J Neurosci 1999 19 3287 97 10212288 10.1523/JNEUROSCI.19-09-03287.1999 PMC6782245

[25] Kawase Y Yanagi Y Takato T Fujimoto M Okochi H Characterization of multipotent adult stem cells from the skin: transforming growth factor-beta (TGF-beta) facilitates cell growth. Exp Cell Res 2004 295 194 203 15051502 10.1016/j.yexcr.2003.12.027

[26] Reynolds BA Weiss S Generation of neurons and astrocytes from isolated cells of the adult mammalian central nervous system. Science 1992 255 1707 10 1553558 10.1126/science.1553558

[27] Uchida S Yokoo S Yanagi Y Usui T Yokota C Mimura T Araie M Yamagami S Amano S Sphere formation and expression of neural proteins by human corneal stromal cells in vitro. Invest Ophthalmol Vis Sci 2005 46 1620 5 15851560 10.1167/iovs.04-0288

[28] Yokoo S Yamagami S Yanagi Y Uchida S Mimura T Usui T Amano S Human corneal endothelial cell precursors isolated by sphere-forming assay. Invest Ophthalmol Vis Sci 2005 46 1626 31 15851561 10.1167/iovs.04-1263

[29] Mimura T Yamagami S Yokoo S Yanagi Y Usui T Ono K Araie M Amano S Sphere therapy for corneal endothelium deficiency in a rabbit model. Invest Ophthalmol Vis Sci 2005 46 3128 35 16123411 10.1167/iovs.05-0251

[30] Mimura T Yokoo S Araie M Amano S Yamagami S Treatment of rabbit bullous keratopathy with precursors derived from cultured human corneal endothelium. Invest Ophthalmol Vis Sci 2005 46 3637 44 16186344 10.1167/iovs.05-0462

[31] Mimura T Yamagami S Yokoo S Araie M Amano S Comparison of rabbit corneal endothelial cell precursors in the central and peripheral cornea. Invest Ophthalmol Vis Sci 2005 46 3645 8 16186345 10.1167/iovs.05-0630

[32] Mimura T Yamagami S Yokoo S Usui T Amano S Selective Isolation of Young Cells from Human Corneal Endothelium by the Sphere-Forming Assay. Tissue Eng Part C Methods 2009 16 803 12 19852617 10.1089/ten.TEC.2009.0608

[33] Pellegrini G Dellambra E Golisano O Martinelli E Fantozzi I Bondanza S Ponzin D McKeon F De Luca M p63 identifies keratinocyte stem cells. Proc Natl Acad Sci USA 2001 98 3156 61 11248048 10.1073/pnas.061032098 PMC30623

[34] Gage FH Mammalian neural stem cells. Science 2000 287 1433 8 10688783 10.1126/science.287.5457.1433

[35] Li H Liu H Heller S Pluripotent stem cells from the adult mouse inner ear. Nat Med 2003 9 1293 9 12949502 10.1038/nm925

[36] Tropepe V Coles BL Chiasson BJ Horsford DJ Elia AJ McInnes RR van der Kooy D Retinal stem cells in the adult mammalian eye. Science 2000 287 2032 6 10720333 10.1126/science.287.5460.2032

[37] Yokoo S Yamagami S Shimada T Usui T Sato TA Amano S Araie M Hamuro J A novel isolation technique of progenitor cells in human corneal epithelium using non-tissue culture dishes. Stem Cells 2008 26 1743 8 18436866 10.1634/stemcells.2007-0866

[38] Qi H Li DQ Shine HD Chen Z Yoon KC Jones DB Pflugfelder SC Nerve growth factor and its receptor TrkA serve as potential markers for human corneal epithelial progenitor cells. Exp Eye Res 2008 86 34 40 17980361 10.1016/j.exer.2007.09.003 PMC2198932

[39] Nakamura T Ang LP Rigby H Sekiyama E Inatomi T Sotozono C Fullwood NJ Kinoshita S The use of autologous serum in the development of corneal and oral epithelial equivalents in patients with Stevens-Johnson syndrome. Invest Ophthalmol Vis Sci 2006 47 909 16 16505023 10.1167/iovs.05-1188

